# Pulmonary Sarcoidosis in Qatar: Clinical Features, Diagnostic Profile and Treatment Patterns in a Multi‐Ethnic Population

**DOI:** 10.1111/crj.70229

**Published:** 2026-09-04

**Authors:** Aasir M. Suliman, Mousa Hussein, Hassan Magboul, Ahmed A. Alsayed, Wisam Alwassiti, Amjad Salman, Saif Alateeg, Amin S. Saied, Mona Allangawi, Hisham A. Sattar

**Affiliations:** ^1^ Pulmonology Department Hamad Medical Corporation Doha Qatar; ^2^ Department of Clinical Medicine, College of Medicine, QU Health Qatar University Doha Qatar; ^3^ Internal Medicine Department Hamad Medical Corporation Doha Qatar

**Keywords:** EBUS‐TBNA, ethnic phenotype, Middle East, Qatar, sarcoidosis

## Abstract

**Introduction:**

Sarcoidosis data from the Middle East are limited, and regional studies have been predominantly single‐ethnicity, leaving phenotyping in the region's diverse expatriate populations unexplored. We characterised pulmonary sarcoidosis across ethnic groups in Qatar and examined temporal trends over 9 years.

**Methods:**

Retrospective cohort study of adults with pulmonary sarcoidosis managed at Hamad Medical Corporation, the principal public‐sector provider in Qatar, between 2015 and 2023. The primary ethnic comparison was Arab versus South Asian. Because diffusion capacity (DLCO) was differentially ascertained, sensitivity analyses assessed the potential impact of missing data.

**Results:**

We included 156 patients (64.7% male; median age 45 years; Arab 45.5%, South Asian 41.0%). Stage II predominated (67.3%); Stage IV was present in 15.4%. Histologic confirmation was achieved in 83.3%, primarily by EBUS‐TBNA (single‐procedure yield 94.3%). Corticosteroids were prescribed in 61.5%. South Asian patients were younger (43 vs. 50 years; *p* = 0.003), more frequently male (80% vs. 54%; *p* = 0.002), and reported constitutional symptoms more often (18.8% vs. 5.6%; *p* = 0.031). Among patients with available measurements, South Asians had lower median DLCO (78.0% vs. 90.5% predicted; *p* = 0.014); however, DLCO was measured in only 48.4% versus 70.4% of South Asian and Arab patients, respectively (*p* = 0.014), limiting interpretation. Asymptomatic presentation increased from 22% in 2015–2019 to 62% in 2020–2021 and 42% in 2022–2023.

**Conclusion:**

In Qatar's first pulmonary sarcoidosis cohort, disease was predominantly Stage II, with high EBUS‐TBNA yield. South Asians had more constitutional symptoms; lower DLCO was observed but requires prospective confirmation. Pandemic‐era imaging may have increased incidental detection.

## Graphical abstract

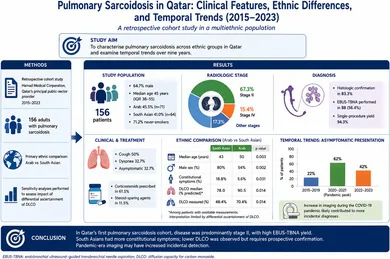

## 1 Introduction

Sarcoidosis is a systemic granulomatous disease of unknown aetiology, characterised by noncaseating granulomas in multiple organs, most commonly the lungs and mediastinal lymph nodes [1, 2]. Its clinical expression is markedly heterogeneous across geographic regions and ethnic populations [3, 4]. Individuals of Black African descent tend to present at a younger age with more severe and multisystem disease [5, 6]; Scandinavian populations exhibit a high incidence of acute presentations such as Löfgren's syndrome [7]; and Japanese cohorts show a distinct phenotype with a high prevalence of cardiac and ocular involvement [8]. Understanding ethnic variation in sarcoidosis therefore has direct implications for clinical recognition and management.

Within the Middle East, a growing body of literature has characterised sarcoidosis in Arab populations, with cohorts from Kuwait [9], Saudi Arabia [10, 11], Oman [12] and Jordan [13], alongside a comprehensive regional review [14]. These studies are almost exclusively composed of Arab nationals and do not capture the substantial non‐Arab populations that characterise many Middle Eastern states—particularly the Gulf, where expatriate communities form the majority of residents.

Qatar offers a unique setting for such an analysis. The country's population exceeds 3.2 million, of whom approximately 88% are expatriates from diverse ethnic backgrounds [15], and all secondary and tertiary public‐sector care is delivered through a single integrated national system—Hamad Medical Corporation (HMC)—providing a unified, systematically documented patient population. Sarcoidosis was previously identified as the most common interstitial lung disease (ILD) in this setting, accounting for 28.3% of a recent 9‐year ILD cohort from the same institution [16]; however, that study did not provide sarcoidosis‐specific phenotyping, ethnic comparisons or temporal trend analyses, and no other dedicated data on pulmonary sarcoidosis from Qatar have been published. The present study describes the clinical features, diagnostic profile and treatment patterns of pulmonary sarcoidosis across ethnic groups managed within this multi‐ethnic tertiary network over 9 years and characterises temporal trends in disease presentation and clinical practice.

## 2 Materials and Methods

### 2.1 Study Design and Setting

This was a retrospective cohort study conducted at HMC, the main public healthcare provider in Qatar. All adult patients with a confirmed diagnosis of pulmonary sarcoidosis identified and followed between January 2015 and December 2023 were included. The cohort was identified from a previously published ILD cohort from Qatar [16], which enrolled 548 consecutive ILD patients across the same study period; the present analysis represents a dedicated examination of the sarcoidosis subgroup. The study was approved by the institutional Medical Research Centre (approval number: MRC‐01‐26‐249; approval date: 21 April 2026), and informed consent was waived in view of the retrospective, anonymised design.

### 2.2 Study Population and Diagnostic Criteria

All adults (≥ 18 years) with a confirmed diagnosis of pulmonary sarcoidosis managed at the centre during the study period were eligible. Diagnosis required compatible clinical and radiological features together with histopathologic evidence of noncaseating granulomas and exclusion of alternative granulomatous diseases, in accordance with the American Thoracic Society (ATS) clinical practice guideline [2].

A minority of patients (26, 16.7%) were diagnosed on clinical and radiological grounds without definitive tissue confirmation, either because tissue sampling was not performed or because the results were inconclusive. Tissue sampling was not performed because the patient declined the procedure, comorbidities precluded the procedure or extrapulmonary sarcoidosis had already been established. In all cases, the diagnosis was established through departmental multidisciplinary review and required the following criteria: (1) a compatible radiological pattern; (2) exclusion of mycobacterial infection based on negative acid‐fast bacilli smear, mycobacterial culture and GeneXpert MTB/RIF testing from sputum and/or bronchoalveolar lavage (BAL), an important consideration given the substantial expatriate population originating from tuberculosis‐endemic regions; (3) no concerning occupational or environmental exposure to an alternative cause of granulomatous lung disease; and (4) a clinical course during follow‐up consistent with sarcoidosis.

### 2.3 Data Collection

Data were extracted retrospectively from the HMC electronic medical records by trained investigators. Variables collected included demographics (age, sex, ethnic group and smoking status); presenting symptoms (cough, dyspnea, chest pain, constitutional symptoms—fever, night sweats or weight loss—arthralgia and skin rash); cardiopulmonary comorbidities; high‐resolution CT (HRCT) findings and Scadding stage; diagnostic procedures and method of definitive diagnosis; BAL differential where performed; pulmonary function tests—forced vital capacity (FVC), FEV_1_ and diffusing capacity for carbon monoxide (DLCO); treatment received; and clinical outcome at last follow‐up. Echocardiography, right heart catheterisation and haemoglobin values were not systematically recorded in the source registry and were therefore unavailable for analysis.

### 2.4 Variable Definitions

#### 2.4.1 Ethnic Groups

Patients were classified into five categories based on nationality and geographic origin: Arab (Qatari and non‐Qatari Arabs combined for analysis given their shared ethnolinguistic background), South Asian, East Asian, African and Caucasian. The primary comparison was between the two largest groups—Arab (*n* = 71) and South Asian (*n* = 64); the remaining groups were reported descriptively.

#### 2.4.2 Scadding Radiological Stage

Scadding stage was derived from HRCT findings [17]: Stage I—bilateral hilar lymphadenopathy (BHL) without parenchymal involvement; Stage II—BHL with parenchymal involvement; Stage III—parenchymal involvement without BHL; and Stage IV—pulmonary fibrosis defined by traction bronchiectasis and/or honeycombing on HRCT, irrespective of lymphadenopathy.

#### 2.4.3 Pulmonary Function Test Pattern

PFT patterns were classified as restrictive (FVC < 80% predicted with FEV_1_/FVC ≥ 0.70, with or without DLCO impairment); obstructive (FEV_1_/FVC < 0.70); isolated DLCO impairment (FVC ≥ 80%, FEV_1_/FVC ≥ 0.70, DLCO < 75%); or normal. Values are reported as percentage predicted using race‐adjusted reference equations applied by the institutional laboratory throughout the study period, in keeping with the ATS/ERS interpretation guidelines prevailing at the time. The 2023 ATS statement subsequently recommended race‐neutral equations [18]; this had not been implemented at our institution during the study period.

### 2.5 Statistical Analysis

Data were analysed using Python (version 3.11). Continuous variables are presented as median with interquartile range (IQR) and categorical variables as counts and percentages. Comparisons between two independent groups used the Mann–Whitney *U* test for continuous variables and Fisher's exact test for categorical variables; comparisons across three or more groups used the Kruskal–Wallis test. Spearman's rank correlation was used to assess monotonic trends. Temporal trends across 2015–2023 were evaluated by Spearman correlation against calendar year on individual‐patient data (*n* = 134) and by epoch comparison between early (2015–2018, *n* = 51) and late (2019–2023, *n* = 83) periods. To examine whether trends reflected pandemic‐related changes in imaging utilisation, an additional stratification compared pre‐COVID (2015–2019), peri‐COVID (2020–2021) and post‐COVID (2022–2023) epochs.

#### 2.5.1 Handling of Missing Pulmonary Function Data

DLCO was not measured in all patients and ascertainment differed by ethnic group. We therefore undertook additional analyses to characterise the potential for selection bias. First, we compared patients with and without DLCO measurement within each ethnic group across age, sex, Scadding Stage IV, symptomatic presentation and corticosteroid prescription. Second, we performed extreme‐case bounding analyses in which all untested patients were assigned, in turn, the most and least favourable possible DLCO status relative to the observed association. Third, we conducted a tipping‐point analysis to determine the impairment prevalence among untested South Asian patients at which the observed association would lose statistical significance. Multivariable linear and logistic regression models including ethnicity, age, sex, ever‐smoking status and Stage IV disease were fitted in complete cases; body mass index and haemoglobin were unavailable. Statistical significance was defined as a two‐tailed *p*‐value < 0.05. No correction was applied for multiple comparisons; comparisons in Table 2 are exploratory and hypothesis‐generating.

## 3 Results

### 3.1 Cohort Overview

Between January 2015 and December 2023, 156 adults with confirmed pulmonary sarcoidosis were identified. Baseline characteristics are summarised in Table 1.

**TABLE 1:** Baseline characteristics of the study cohort (*n* = 156).

Characteristic	*n* (%) or median (IQR)
Demographics	
Total patients	156
Age, years—median (IQR)	45.0 (38.0–55.0)
Male sex	101 (64.7)
Female sex	55 (35.3)
Ethnic group	
Arab—non‐Qatari	64 (41.0)
Arab—Qatari	7 (4.5)
South Asian	64 (41.0)
East Asian	7 (4.5)
African	6 (3.8)
Caucasian	8 (5.1)
Smoking status	
Never smoker	111 (71.2)
Current smoker	25 (16.0)
Ex‐smoker	20 (12.8)
Clinical presentation	
Asymptomatic (incidental finding)	51 (32.7)
Cough	78 (50.0)
Dyspnea	51 (32.7)
Chest pain	19 (12.2)
Constitutional symptoms	19 (12.2)
Arthralgia	21 (13.5)
Skin rash	15 (9.6)
Scadding stage	
Stage I	23 (14.7)
Stage II	105 (67.3)
Stage III	4 (2.6)
Stage IV	24 (15.4)
HRCT findings	
Mediastinal lymphadenopathy	150 (96.2)
Pulmonary nodules	110 (70.5)
Ground‐glass opacity	43 (27.6)
Septal thickening/reticulation	39 (25.0)
Traction bronchiectasis	23 (14.7)
Consolidation	24 (15.4)
Honeycombing	3 (1.9)
Diagnostic procedures performed	
EBUS‐TBNA	88 (56.4)
Transbronchial lung biopsy (TBLB)	34 (21.8)
Bronchoalveolar lavage (BAL)	44 (28.2)
CT‐guided biopsy	5 (3.2)
Surgical biopsy (VATS/thoracotomy)	15 (9.6)
Primary method of diagnosis	
EBUS‐TBNA	83 (53.2)
TBLB	28 (17.9)
Clinical diagnosis	26 (16.7)
VATS	10 (6.4)
CT‐guided biopsy	5 (3.2)
Thoracotomy	4 (2.6)
Pulmonary function tests	
PFTs performed	95 (60.9)
FVC % predicted—median (IQR)	82.0 (74.0–95.0)
FEV_1_ % predicted—median (IQR)	81.0 (73.5–93.0)
DLCO % predicted—median (IQR)	83.0 (74.0–97.0)
PFT pattern	
Normal	49 (51.6)
Restrictive	36 (37.9)
Obstructive	3 (3.2)
Isolated DLCO impairment	7 (7.4)
Treatment^a^	
Systemic corticosteroids	96 (61.5)
No treatment (watchful waiting)	59 (37.8)
Steroid‐sparing monotherapy (methotrexate)	1 (0.6)
Methotrexate (any line)	10 (6.4)
Mycophenolate mofetil	6 (3.8)
Azathioprine	3 (1.9)
Outcome at last encounter	
Alive	96 (61.5)
Lost to follow‐up	59 (37.8)
Deceased	1 (0.6)

Abbreviations: BAL, bronchoalveolar lavage; DLCO, diffusing capacity for carbon monoxide; EBUS‐TBNA, endobronchial ultrasound‐guided transbronchial needle aspiration; FEV_1_, forced expiratory volume in 1 s; FVC, forced vital capacity; HRCT, high‐resolution computed tomography; IQR, interquartile range; LN, lymph node; TBLB, transbronchial lung biopsy; VATS, video‐assisted thoracoscopic surgery.

^a^ One patient received methotrexate as first‐line monotherapy and is not counted in either the corticosteroid or watchful‐waiting category. One patient received methotrexate followed sequentially by mycophenolate mofetil and is counted once in the steroid‐sparing total of 18 patients (11.5%).

### 3.2 Demographics and Clinical Presentation

The cohort was predominantly male (*n* = 101, 64.7%), with a median age at diagnosis of 45.0 years (IQR: 38.0–55.0; range: 29–77). Arab patients constituted the largest group (*n* = 71, 45.5%), followed by South Asians (*n* = 64, 41.0%), Caucasians (*n* = 8, 5.1%), East Asians (*n* = 7, 4.5%) and Africans (*n* = 6, 3.8%). Most were never‐smokers (*n* = 111, 71.2%), and only 25 patients (16.0%) had a relevant respiratory or cardiovascular comorbidity; asthma was the most prevalent (*n* = 17, 10.9%).

At presentation, 51 patients (32.7%) were entirely asymptomatic, representing incidental radiological findings. Among the 105 symptomatic patients, cough was most common (*n* = 78, 50.0%), followed by dyspnea (*n* = 51, 32.7%), arthralgia (*n* = 21, 13.5%), chest pain and constitutional symptoms (*n* = 19 each, 12.2%) and skin rash (*n* = 15, 9.6%).

### 3.3 Diagnostic Profile

Stage II was the predominant Scadding stage (67.3%), followed by Stage IV (15.4%), Stage I (14.7%) and Stage III (2.6%). HRCT features and procedure counts are detailed in Table 1. Histologic confirmation was achieved in 130 patients (83.3%). EBUS‐TBNA was the primary diagnostic method in 83 of the 88 cases in which it was performed (procedure yield 94.3%) and the dominant primary modality overall (53.2%); a single procedure was sufficient in 94% of histologically confirmed cases.

BAL was performed in 44 patients (28.2%) to exclude alternative aetiologies, not as a primary diagnostic method. The differential was macrophage‐predominant (median: 71.5%, IQR: 49.2–84.2), with median lymphocytes 10.5% (IQR: 7.8–19.2) and lymphocytosis ≥ 15% in 16 patients (36.4%). Strong inverse correlations were observed between lymphocyte and macrophage percentages (*r* = −0.539, *p* < 0.001) and between neutrophil and macrophage percentages (*r* = −0.670, *p* < 0.001). BAL cell fractions did not correlate with FVC or DLCO and did not differ across Scadding stages or ethnic groups.

PFTs were performed in 95 patients (60.9%), including DLCO measurements in 81 (52.0% of the total cohort). Median FVC was 82.0% predicted (IQR: 74.0–95.0), FEV_1_ 81.0% (73.5–93.0) and DLCO 83.0% (74.0–97.0). The ventilatory pattern was normal in 49 patients (51.6%), restrictive in 36 (37.9%), with isolated DLCO impairment in seven (7.4%) and obstructive in three (3.2%).

### 3.4 Treatment and Outcomes

Systemic corticosteroids were prescribed in 96 patients (61.5%), and 59 (37.8%) were managed with watchful waiting; one patient received methotrexate as first‐line monotherapy without corticosteroids. Steroid‐sparing agents were used in 18 patients (11.5%): methotrexate (*n* = 10, 6.4%), mycophenolate mofetil (*n* = 6, 3.8%) and azathioprine (*n* = 3, 1.9%). At the last encounter, 96 patients (61.5%) were alive and under active follow‐up, 59 (37.8%) had been lost to follow‐up and one (0.6%) had died.

### 3.5 Ethnic Group Comparison

A detailed comparison between Arab (*n* = 71) and South Asian (*n* = 64) patients was performed; the East Asian, African and Caucasian groups are reported descriptively. Full data are presented in Table 2.

**TABLE 2:** Comparison of clinical phenotype across ethnic groups.

Characteristic	Arab (*n* = 71)	South Asian (*n* = 64)	East Asian (*n* = 7)	African (*n* = 6)	Caucasian (*n* = 8)	*p* ^a^
Demographics						
Age, years—median (IQR)	50.0 (40.5–59.0)	43.0 (36.0–51.2)	43.0 (40.5–53.0)	42.5 (38.8–47.0)	53.0 (45.8–63.2)	0.003**
Male sex	38 (53.5%)	51 (79.7%)	3 (42.9%)	6 (100.0%)	3 (37.5%)	0.002**
Clinical presentation (complete ascertainment)						
Asymptomatic	25 (35.2%)	20 (31.2%)	3 (42.9%)	1 (16.7%)	2 (25.0%)	0.715
Cough	31 (43.7%)	33 (51.6%)	4 (57.1%)	5 (83.3%)	5 (62.5%)	0.391
Dyspnea	28 (39.4%)	16 (25.0%)	2 (28.6%)	2 (33.3%)	3 (37.5%)	0.098
Chest pain	10 (14.1%)	5 (7.8%)	1 (14.3%)	0 (0.0%)	3 (37.5%)	0.284
Constitutional symptoms	4 (5.6%)	12 (18.8%)	0 (0.0%)	2 (33.3%)	1 (12.5%)	0.031*
Arthralgia	8 (11.3%)	12 (18.8%)	1 (14.3%)	0 (0.0%)	0 (0.0%)	0.237
Skin rash	7 (9.9%)	5 (7.8%)	1 (14.3%)	1 (16.7%)	1 (12.5%)	0.768
Scadding stage						
Stage I	11 (15.5%)	9 (14.1%)	3 (42.9%)	0 (0.0%)	0 (0.0%)	1.000
Stage II	49 (69.0%)	44 (68.8%)	3 (42.9%)	4 (66.7%)	5 (62.5%)	1.000
Stage III	0 (0.0%)	3 (4.7%)	0 (0.0%)	0 (0.0%)	1 (12.5%)	0.104
Stage IV	11 (15.5%)	8 (12.5%)	1 (14.3%)	2 (33.3%)	2 (25.0%)	0.805
Pulmonary function (incomplete ascertainment—see Table 3)						
DLCO measured, *n* (% of group)	50 (70.4%)	31 (48.4%)	6 (85.7%)	2 (33.3%)	4 (50.0%)	0.014*
FVC %—median (IQR)	83.5 (76.2–96.8)	79.0 (73.0–88.0)	95.0 (92.5–99.5)	74.0 (73.0–88.0)	79.0 (78.0–85.0)	0.098
DLCO %—median (IQR)	90.5 (78.5–98.8)	78.0 (69.5–84.5)	103.0 (94.0–109.8)	82.0 (81.5–82.5)	80.0 (76.0–83.0)	0.014*
DLCO < 75% predicted, *n*/*N* tested (%)	10/50 (20.0)	13/31 (41.9)	0/6 (0.0)	0/2 (0.0)	1/4 (25.0)	0.044*
Complete‐case multivariable analysis^b^						
Adjusted Δ DLCO % pred (95% CI)	Reference	−10.8 (−19.7 to −2.0)	—	—	—	0.017*
Adjusted OR for DLCO < 75% (95% CI)	Reference	3.98 (1.24–12.73)	—	—	—	0.020*

*Note:* Values are *n* (%) or median (IQR).

^a^ *p*‐value: Arab vs. South Asian comparison. Categorical: Fisher's exact test. Continuous: Mann–Whitney *U* test. Other groups descriptive only.

^b^ Models include ethnicity, age, sex, ever‐smoking and Stage IV disease; complete cases *n* = 81 (Arab: 50, South Asian: 31). BMI and haemoglobin unavailable. Because DLCO ascertainment differed significantly by ethnicity, these estimates are subject to potential selection bias and should be interpreted alongside the sensitivity analyses in Table 3.

* *p* < 0.05.

** *p* < 0.01.

#### 3.5.1 Demographics

Arab patients were older at diagnosis than South Asian patients (median: 50.0 vs. 43.0 years; *p* = 0.003) and less frequently male (54% vs. 80%; *p* = 0.002).

#### 3.5.2 Clinical Presentation

Constitutional symptoms—ascertained in all 135 patients in the primary comparison—were significantly more frequent in South Asian than Arab patients (12/64, 18.8% vs. 4/71, 5.6%; *p* = 0.031). There was a nonsignificant trend toward higher dyspnea rates in Arab patients (39% vs. 25%; *p* = 0.098). Asymptomatic presentation was comparable (35% vs. 31%; *p* = 0.715).

#### 3.5.3 Scadding Stage

The radiological stage distribution was comparable, with Stage II predominating in both (69.0% vs. 68.8%), and Stage IV present in 15.5% and 12.5%, respectively (*p* = 0.805).

#### 3.5.4 Pulmonary Function and the Effect of Differential Ascertainment

DLCO was measured in 50 of 71 Arab patients (70.4%) but only 31 of 64 South Asian patients (48.4%); this difference in ascertainment was itself statistically significant (*p* = 0.014). Among patients tested, South Asians had a lower median DLCO (78.0% vs. 90.5% predicted; *p* = 0.014) and a higher prevalence of DLCO < 75% predicted (13/31, 41.9% vs. 10/50, 20.0%; *p* = 0.044), despite comparable Scadding stage and FVC (median: 79.0% vs. 83.5%; *p* = 0.098). In complete‐case multivariable models adjusting for age, sex, ever‐smoking and Stage IV disease (*n* = 81), South Asian ethnicity was associated with a 10.8 percentage‐point lower DLCO (95% CI: −19.7 to −2.0; *p* = 0.017) and with DLCO < 75% predicted (adjusted OR: 3.98, 95% CI: 1.24–12.73; *p* = 0.020).

#### 3.5.5 Sensitivity Analyses for Missing DLCO Data

Patients with and without DLCO measurement did not differ significantly within either ethnic group on any recorded characteristic, including age, sex, Stage IV disease, symptomatic presentation or corticosteroid prescription (all *p* ≥ 0.13; Table 3). Under a missing‐at‐random assumption, in which untested patients were assigned the impairment prevalence observed among tested patients of the same ethnicity, the association persisted (42.2% vs. 19.7%; *p* = 0.005). Tipping‐point analysis indicated that the association would lose statistical significance if fewer than 33% of the 33 untested South Asian patients were impaired, compared with 41.9% among those tested. However, under extreme‐case bounding—assigning impairment to all 21 untested Arab patients and normal DLCO to all 33 untested South Asian patients—the direction of the association reversed (43.7% vs. 20.3%; *p* = 0.006). The observed DLCO difference is therefore compatible with, but not established by, the available data and is reported as hypothesis‐generating.

**TABLE 3:** Sensitivity analyses for differential DLCO ascertainment (Arab vs. South Asian).

Panel A. Comparison of patients with and without DLCO measurement, within ethnic group.						
Characteristic	Arab tested (*n* = 50)	Arab untested (*n* = 21)	*p*	South Asian tested (*n* = 31)	South Asian untested (*n* = 33)	*p*
Age, years—median	50	47	0.129	45	39	0.194
Male sex	26 (52%)	12 (57%)	0.796	23 (74%)	28 (85%)	0.359
Scadding Stage IV	10 (20%)	1 (5%)	0.156	5 (16%)	3 (9%)	0.468
Symptomatic at presentation	30 (60%)	11 (52%)	0.605	22 (71%)	22 (67%)	0.791
Systemic corticosteroids	33 (66%)	14 (67%)	1.000	19 (61%)	18 (55%)	0.621

Panel B. Missing‐data scenarios for DLCO < 75% predicted.			
Scenario	Arab, *n*/*N* (%)	South Asian, *n*/*N* (%)	*p*
Complete case (observed)	10/50 (20.0)	13/31 (41.9)	0.044
Missing at random^a^	14/71 (19.7)	27/64 (42.2)	0.005
Extreme case favouring the association^b^	10/71 (14.1)	46/64 (71.9)	< 0.001
Extreme case opposing the association^c^	31/71 (43.7)	13/64 (20.3)	0.006 (reversed)

^a^ Untested patients assigned the impairment prevalence observed among tested patients of the same ethnicity.

^b^ All untested South Asian patients assumed impaired; all untested Arab patients assumed normal.

^c^ All untested Arab patients assumed impaired; all untested South Asian patients assumed normal. Tipping‐point analysis: The association loses statistical significance if fewer than 33% of untested South Asian patients are impaired (observed prevalence among tested South Asian patients, 41.9%).

### 3.6 Temporal Trends (2015–2023)

**FIGURE 1:**
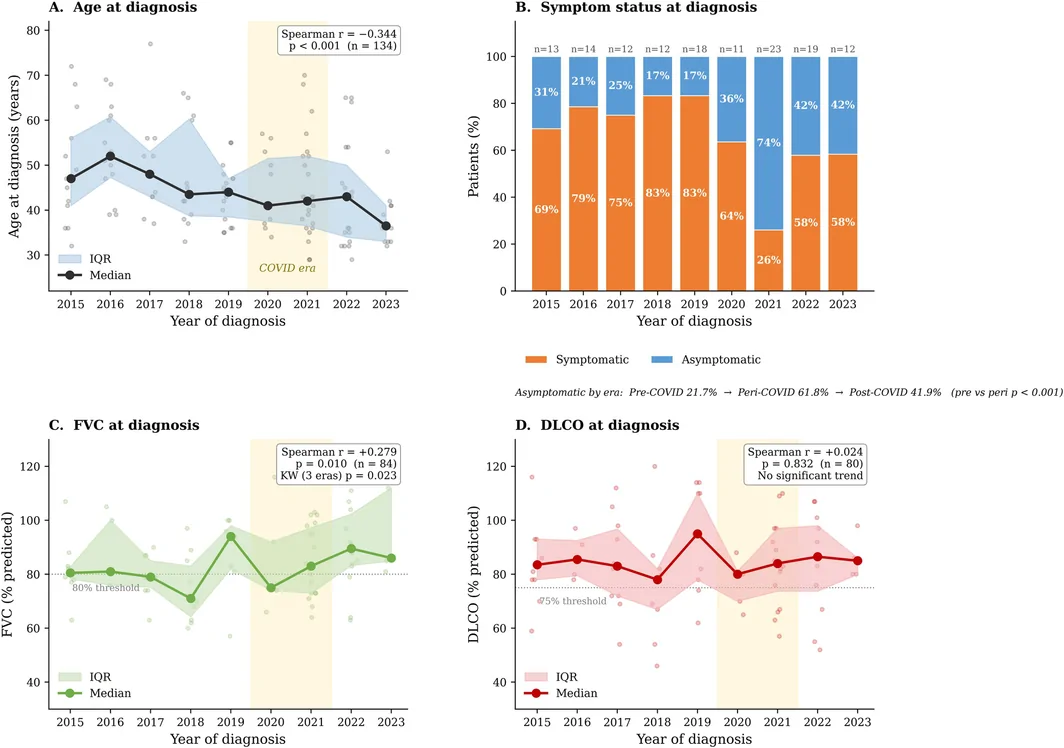
Temporal trends in patient and disease characteristics at presentation (2015–2023, *n* = 134). The shaded vertical band in Panels A, C and D denotes the peri‐COVID period (2020–2021). (A) Age at diagnosis: median (line), interquartile range (band), individual patients (dots); Spearman *r* = −0.344, *p* < 0.001. (B) Symptom status: 100% stacked bars; asymptomatic presentation rose from 21.7% pre‐COVID (2015–2019) to 61.8% peri‐COVID (2020–2021) and 41.9% post‐COVID (2022–2023); pre vs. peri, *p* < 0.001. (C) FVC % predicted: Spearman *r* = +0.279, *p* = 0.010 (*n* = 84), with stepwise rise across the pre/peri/post‐COVID stratification (Kruskal–Wallis, *p* = 0.023); dashed line marks the 80% impairment threshold. (D) DLCO % predicted: no significant temporal trend (*r* = +0.024, *p* = 0.832, *n* = 80); dashed line marks the 75% impairment threshold.

**FIGURE 2:**
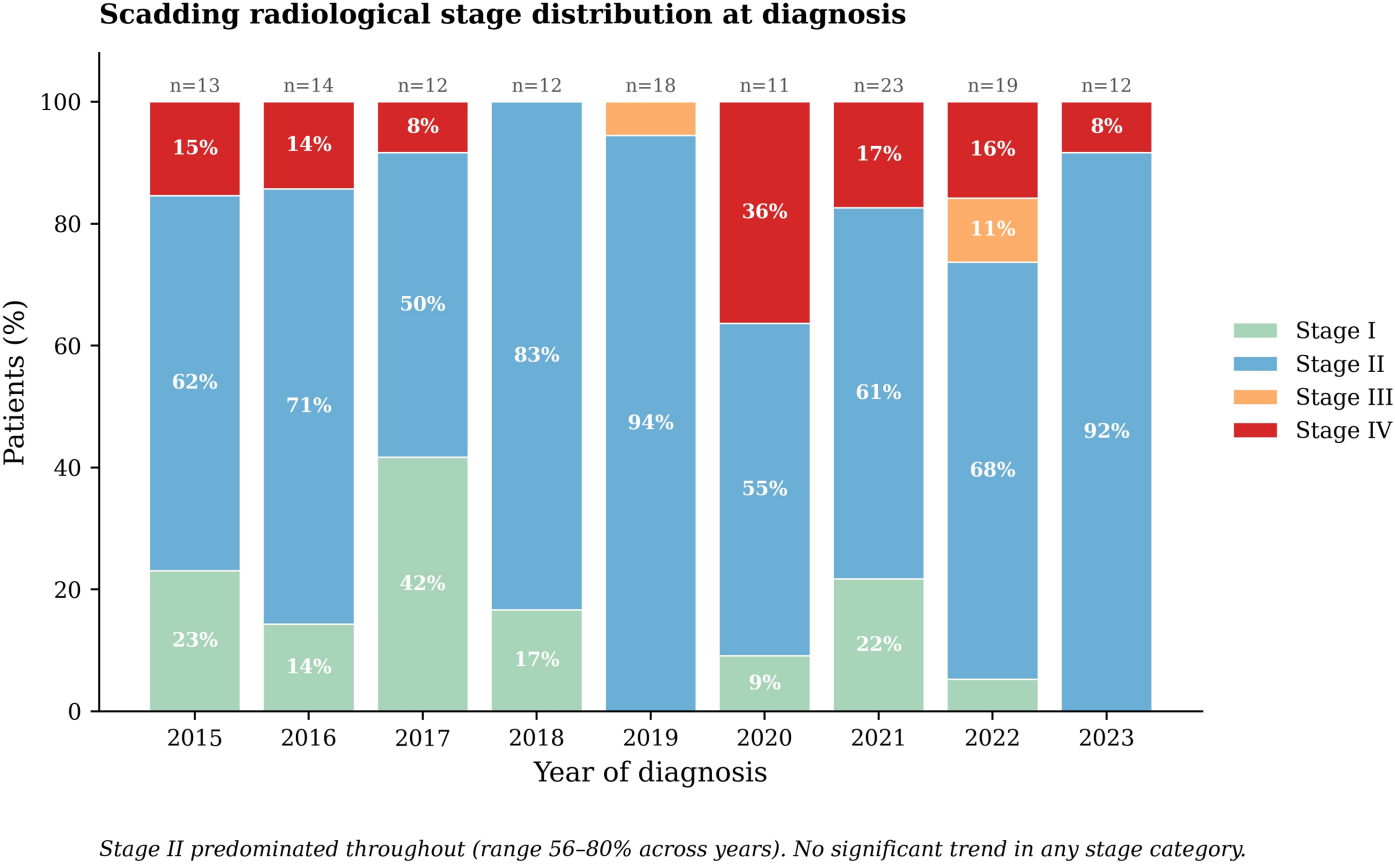
Scadding radiological stage distribution at diagnosis by year (2015–2023, *n* = 134), shown as 100% stacked bars with the within‐year proportion of each stage. Stage II predominated throughout (range 56%–80% across years); no stage category showed a significant trend over the study period. Annual case counts are shown above each bar.

**FIGURE 3:**
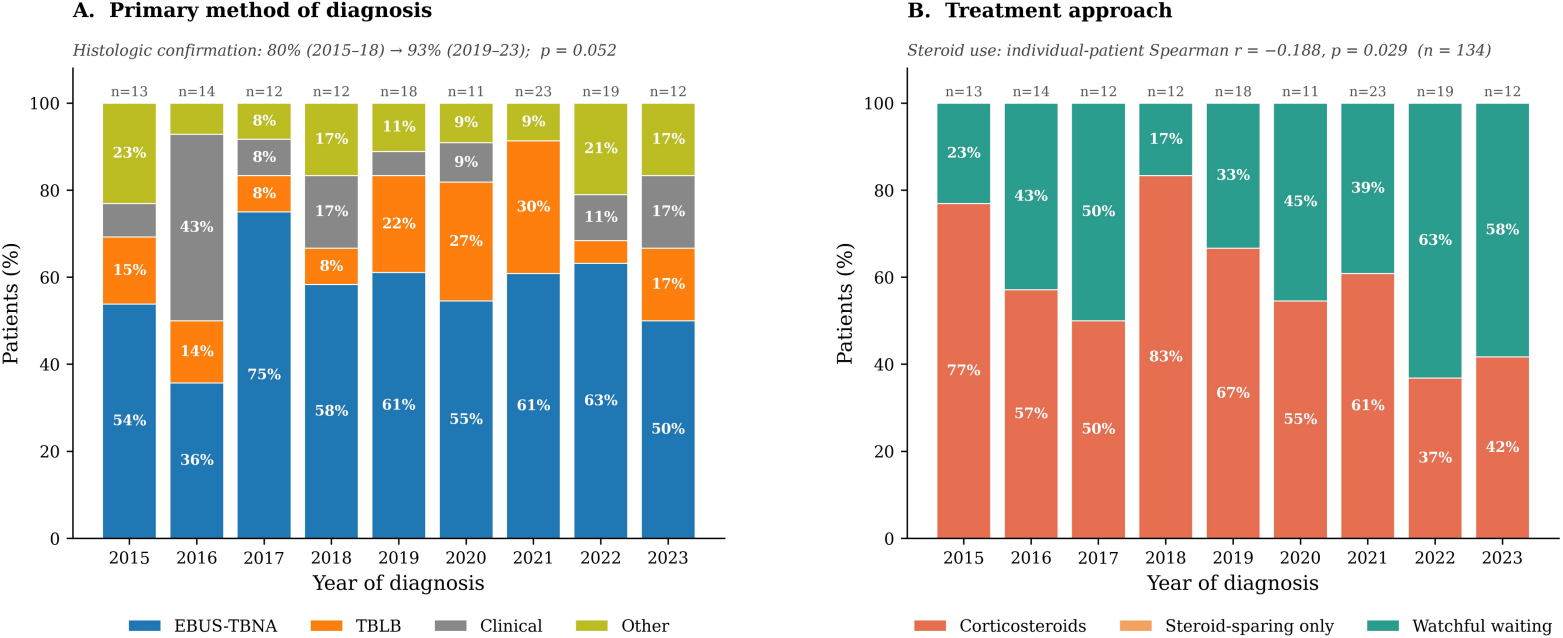
Diagnostic and treatment practice over time (2015–2023, *n* = 134). (A) Primary method of diagnosis: Histologic confirmation rose from 80% (2015–2018) to 93% (2019–2023) (*p* = 0.052), with corresponding decline in clinical diagnosis; EBUS‐TBNA remained the dominant primary modality. (B) Treatment approach: systemic corticosteroid prescription declined (Spearman *r* = −0.188, *p* = 0.029) with a parallel rise in watchful waiting.

Temporal trends were evaluated on individual‐patient data restricted to patients diagnosed within the study window (*n* = 134) and are illustrated in Figures 1, 2, 3.

#### 3.6.1 Demographics

A significant decline in age at diagnosis was observed (Spearman *r* = −0.344, *p* < 0.001), with the median falling from 48 years in 2015–2018 to 41 years in 2019–2023 (Figure 1A).

#### 3.6.2 Clinical Presentation

Asymptomatic presentation rose across the study period (*r* = +0.228, *p* = 0.008). COVID‐era stratification clarified the pattern: 15/69 (21.7%) pre‐COVID (2015–2019), 21/34 (61.8%) peri‐COVID (2020–2021) and 13/31 (41.9%) post‐COVID (2022–2023) (pre vs. peri, *p* < 0.001; pre vs. post, *p* = 0.054). Dyspnea at presentation fell from 47% to 19% (*p* < 0.001) (Figure 1B).

#### 3.6.3 Scadding Stage

No significant trend was observed in stage distribution; Stage II remained predominant throughout (Figure 2).

#### 3.6.4 Method of Diagnosis

Histologic confirmation rose from 80% in 2015–2018 to 93% in 2019–2023 (*p* = 0.052). EBUS‐TBNA remained the dominant primary modality, whereas TBLB use increased (14% vs. 27%; *p* = 0.089) (Figure 3A).

#### 3.6.5 Pulmonary Function

Median FVC at diagnosis rose across the study window (*r* = +0.279, *p* = 0.010), with a stepwise increase across the COVID stratification (79.5% pre‐COVID, 82.0% peri‐COVID, 88.0% post‐COVID; Kruskal–Wallis *p* = 0.023). No significant trend was observed for DLCO (*r* = +0.024, *p* = 0.83) (Figure 1C,D).

#### 3.6.6 Treatment

Steroid prescription declined (*r* = −0.188, *p* = 0.029) with a parallel rise in watchful waiting, from 67% (2015–2018) to 53% (2019–2023) (*p* = 0.150) (Figure 3B).

## 4 Discussion

This study reports the clinical features, diagnostic profile and treatment patterns of pulmonary sarcoidosis in 156 patients across five ethnic groups managed within a unified tertiary healthcare network in Qatar over a 9‐year period. To our knowledge, this represents the largest dedicated sarcoidosis cohort from Qatar and one of the largest multi‐ethnic series from the Middle East.

### 4.1 Clinical and Demographic Profile

The male predominance (64.7%) contrasts with the female predominance reported across Middle Eastern series—from 57% female in Kuwait [9] to 72% in Oman [12]—and with the global pattern in which women are more frequently affected [1, 19]. This divergence is unlikely to reflect a biological sex difference and is more likely explained by Qatar's demographic structure, in which working‐age male expatriates form a disproportionately large segment of the population; South Asian patients, accounting for 41% of the cohort, were 80% male. Similarly, the younger age at diagnosis among South Asian patients most plausibly reflects the age structure of the migrant labour force rather than an ethnic difference in disease onset.

### 4.2 Diagnostic Profile

Stage II disease predominated (67.3%), higher than the broader Middle Eastern experience (17%–61%) [14]. The Stage IV prevalence of 15.4% is notably higher than 1.8% in Kuwait [9] and 12.5% in Saudi Arabia [10], suggesting a more advanced disease burden at presentation; this likely reflects tertiary‐centre referral patterns rather than a true difference in disease biology.

Histologic confirmation was achieved in 83.3%, and EBUS‐TBNA yield (94.3%) slightly exceeded the published range of 54%–93% [20, 21], supporting the adequacy of a single‐procedure approach where EBUS‐TBNA is available. The reduction in clinical diagnosis from 20% to 7% across the study period likely reflects broader adoption of TBLB to complement EBUS‐TBNA and a lower threshold for histologic confirmation given the substantial expatriate population from tuberculosis‐endemic regions.

### 4.3 Differences in Disease Phenotype Between Arab and South Asian Patients

The clearest observed difference between Arab and South Asian patients was in constitutional symptoms, which were more than three times as frequent among South Asian patients (18.8% vs. 5.6%; *p* = 0.031). This finding is consistent with international observations that minority ethnic groups may present with more symptomatic and multisystem disease [6] and may reflect delayed presentation, differences in symptom reporting or underlying biological variation.

The apparent divergence in DLCO between Arab and South Asian patients warrants cautious interpretation, particularly given the differential ascertainment of pulmonary function testing. DLCO was measured in 48.4% of South Asian patients compared with 70.4% of Arab patients. Care at HMC is delivered through a single public‐sector healthcare network, and all residents of Qatar, including expatriate workers, are eligible for subsidised care through the national health card system; thus, testing is not primarily determined by insurance status. Nevertheless, unmeasured structural and socio‐economic factors—including employment‐related constraints, shift patterns, transportation, language concordance, health literacy and population mobility—may have contributed to differential ascertainment, although these factors were not directly assessed in the present study. Importantly, tested and untested patients did not differ detectably across the recorded clinical characteristics, and the association between South Asian patients and lower DLCO persisted after multivariable adjustment and under a missing‐at‐random assumption. Extreme‐case bounding further showed that reversal of the observed association would require the prevalence of DLCO impairment among untested South Asian patients to be approximately 33%, compared with 41.9% among those who were tested. These analyses provide some reassurance that differential ascertainment alone may not fully account for the observed association, although unmeasured differences between tested and untested patients cannot be excluded.

Additional factors may influence DLCO and could not be fully accounted for in this retrospective cohort include the absence of body mass index and haemoglobin data, both of which may influence measured DLCO, and the use of race‐adjusted reference equations; replication using the 2023 ATS race‐neutral approach [18] would therefore be valuable. Pulmonary vascular involvement has also been proposed as a potential contributor to disproportionate DLCO reduction [22, 23], but systematic echocardiographic or haemodynamic assessment was not performed in this cohort, precluding evaluation of this mechanism. Overall, the observed difference in DLCO should therefore be regarded as a hypothesis‐generating association requiring prospective confirmation with systematic pulmonary function testing and standardised, race‐neutral interpretation.

### 4.4 Temporal Trends and the COVID‐19 Effect

Three trends over the study period describe a shift in the clinical phenotype of newly diagnosed sarcoidosis in Qatar: declining age at diagnosis, rising asymptomatic presentation and improving baseline FVC. The asymptomatic presentation trend was not linear but markedly clustered around the pandemic years: Rates rose from 22% pre‐COVID to 62% during 2020–2021 and partially regressed to 42% in 2022–2023, with median FVC following the same pattern. The most parsimonious interpretation is that widespread chest CT performed for nonrespiratory and COVID‐related indications during 2020–2021 detected a wave of mild, incidentally diagnosed sarcoidosis that partially reverted as imaging practice normalised, consistent with Western reports of rising incidental sarcoidosis diagnosis on CT [24]. Steroid prescription declined across the same period (*r* = −0.188, *p* = 0.029), reflecting the increasing proportion of asymptomatic patients for whom treatment is not indicated by current guidelines [25].

### 4.5 Limitations

The key limitation of this study is the incomplete and differential ascertainment of pulmonary function testing, which limits interpretation of the observed ethnic difference in DLCO, as discussed above. The retrospective design carries the risk of incomplete data capture, and the analysis was restricted to pulmonary sarcoidosis without systematic assessment of extrapulmonary manifestations. The 37.8% loss to follow‐up precluded long‐term outcome analysis. The small sample sizes of the East Asian, African and Caucasian subgroups limit comparisons beyond the two main groups. With only one death, survival analysis was not feasible. BMI, haemoglobin and echocardiography were unavailable. Finally, the temporal trend analysis is observational and cannot directly attribute the asymptomatic presentation surge to COVID‐related imaging, although the temporal clustering is strongly suggestive.

## 5 Conclusions

In this largest multi‐ethnic cohort of pulmonary sarcoidosis from Qatar, the cohort was predominantly male, reflecting the demographic characteristics of Qatar's expatriate workforce. Disease presented predominantly at Scadding Stage II and was diagnosed with a high single‐procedure yield of EBUS‐TBNA. South Asian patients presented more frequently with constitutional symptoms than Arab patients. A lower diffusing capacity was observed among South Asian patients; however, differential ascertainment of pulmonary function testing limits interpretation of this finding and warrants prospective confirmation with systematic physiological assessment. A pandemic‐era increase in incidentally detected, milder disease accounted for much of the apparent temporal shift toward asymptomatic presentation. These findings provide a regional benchmark for pulmonary sarcoidosis and highlight the value of ethnicity‐aware phenotyping alongside standardised physiological assessment.

## Author Contributions

A.M.S. made substantial contributions to the conception and design of the work, was responsible for data curation, formal analysis and investigation and drafted the original manuscript. M.H. contributed significantly to methodology, formal analysis and supervision, and provided critical revision of the manuscript. H.A.S. and M.A. provided senior supervision and contributed to investigation and methodology. H.M., A.A.A., W.A., A.S., S.A. and A.S.S. contributed to data acquisition, methodology and interpretation of results. All authors substantially contributed to the revision of the manuscript drafts. All authors meet the ICMJE authorship criteria, have approved the submitted version of the manuscript and have agreed to be accountable for all aspects of the work.

## Funding

The authors acknowledge Qatar National Library for providing financial support for open‐access publication of this article.

## Ethics Statement

The study was approved by the institutional Medical Research Centre (approval number MRC‐01‐26‐249; approval date 21 April 2026), and informed consent was waived in view of the retrospective, anonymised design.

## Conflicts of Interest

The authors declare no conflicts of interest.

## Data Availability

The data that support the findings of this study are available from the corresponding author upon reasonable request. Data are not publicly available because they contain information that could compromise the privacy of research participants and are subject to institutional governance by Hamad Medical Corporation.
